# Pathogenesis of Enamel-Renal Syndrome Associated Gingival Fibromatosis: A Proteomic Approach

**DOI:** 10.3389/fendo.2021.752568

**Published:** 2021-10-29

**Authors:** Victor Simancas Escorcia, Clément Guillou, Lilia Abbad, Louise Derrien, Claudio Rodrigues Rezende Costa, Vidjea Cannaya, Mourad Benassarou, Christos Chatziantoniou, Ariane Berdal, Ana Carolina Acevedo, Olivier Cases, Pascal Cosette, Renata Kozyraki

**Affiliations:** ^1^Centre de Recherche des Cordeliers, Sorbonne Université, INSERM, Université de Paris, Oral Molecular Pathophysiology, Paris, France; ^2^Normandie Université, PISSARO Proteomic Facility, Institute for Research and Innovation in Biomedicine (IRIB), Mont-Saint-Aignan, France; ^3^Normandie Université, UMR670 Centre National de la Recherche Scientifique (CNRS), Mont-Saint-Aignan, France; ^4^UMRS1155, INSERM, Sorbonne Université, Paris, France; ^5^Oral Center for Inherited Diseases, University Hospital of Brasília, Oral Histopathology Laboratory, Department of Dentistry, Health Sciences Faculty, University of Brasília (UnB), Brasília, Brazil; ^6^Service de Chirurgie Maxillo-faciale et Stomatologie, Hôpital De la Pitié Salpétrière, Sorbonne Université, Paris, France; ^7^Centre de Référence Maladies Rares (CRMR) O-RARES, Hôpital Rothshild, Unité de Formation et de Recherche (UFR) d’Odontologie-Garancière, Université de Paris, Paris, France

**Keywords:** FAM20A, FAM20C, secretome analysis, enamel renal syndrome, gingival fibroblast, gingival fibromatosis, fibrosis, TGF-beta

## Abstract

The enamel renal syndrome (ERS) is a rare disorder featured by amelogenesis *imperfecta*, gingival fibromatosis and nephrocalcinosis. ERS is caused by bi-allelic mutations in the secretory pathway pseudokinase FAM20A. How mutations in *FAM20A* may modify the gingival connective tissue homeostasis and cause fibromatosis is currently unknown. We here analyzed conditioned media of gingival fibroblasts (GFs) obtained from four unrelated ERS patients carrying distinct mutations and control subjects. Secretomic analysis identified 109 dysregulated proteins whose abundance had increased (69 proteins) or decreased (40 proteins) at least 1.5-fold compared to control GFs. Proteins over-represented were mainly involved in extracellular matrix organization, collagen fibril assembly, and biomineralization whereas those under-represented were extracellular matrix-associated proteins. More specifically, transforming growth factor-beta 2, a member of the TGFβ family involved in both mineralization and fibrosis was strongly increased in samples from GFs of ERS patients and so were various known targets of the TGFβ signaling pathway including Collagens, Matrix metallopeptidase 2 and Fibronectin. For the over-expressed proteins quantitative RT-PCR analysis showed increased transcript levels, suggesting increased synthesis and this was further confirmed at the tissue level. Additional immunohistochemical and western blot analyses showed activation and nuclear localization of the classical TGFβ effector phospho-Smad3 in both ERS gingival tissue and ERS GFs. Exposure of the mutant cells to TGFB1 further upregulated the expression of TGFβ targets suggesting that this pathway could be a central player in the pathogenesis of the ERS gingival fibromatosis.

In conclusion our data strongly suggest that TGFβ -induced modifications of the extracellular matrix contribute to the pathogenesis of ERS. To our knowledge this is the first proteomic-based analysis of FAM20A-associated modifications.

## Introduction

The Enamel Renal Syndrome (ERS) is a rare autosomal recessive disorder caused by loss-of-function mutations in the FAM20A Golgi associated secretory pathway pseudokinase gene (OMIM#611062). ERS is characterized by amelogenesis *imperfecta* (AI), impaired tooth eruption and gingival fibromatosis. Ectopic accumulation of calcium/phosphate complexes, within gingival, pulpal and renal tissues are typically associated with the disease (1–8).

FAM20A and the other two members of the family with sequence similarity 20, FAM20B and FAM20C, were initially identified in hematopoietic cells. FAM20B is a xylosyl-kinase that phosphorylates xylose residues on conserved glycosaminoglycan-protein linkage regions of proteoglycans (9). Compound heterozygous mutations in *FAM20B* are believed to cause lethal short limb dysplasia (10). FAM20C is the Golgi associated secretory pathway kinase responsible for phosphorylating most of the secreted phosphoproteins on the SxE/pS motif. Loss of function mutations in the *FAM20C* gene cause the Raine syndrome (RS, OMIM#259775), a rare autosomal recessive disorder, generally leading to a lethal osteosclerotic bone dysplasia. In non-lethal RS forms, hypophosphatemic rickets, neurological disorders, amelogenesis *imperfecta* (AI) and gingival overgrowth were reported (11). FAM20A is a secreted pathway pseudokinase strongly expressed in dental matrices and gingival fibroblasts (8, 12). *In vitro* FAM20A forms a complex with FAM20C and promotes its kinase activity (13). Although the *in vivo* function of FAM20A remains elusive it is interesting to note that *Fam20A* inactivation in the mouse has been associated with calcifications of muscular arteries in various organs such as heart and kidney (14).

We previously showed that, in addition to calcium deposits, the ERS gingival connective tissue contained increased amounts of disorganized collagen fiber bundles and abnormally expressed proteoglycans of the heparan and keratan sulfate families including Aggrecan, a major cartilage component. Additionally, Periostin (POSTN) which modulates the expression of collagen, Aggrecan and other extracellular matrix (ECM) components was abnormally distributed throughout the ERS gingival interstitium (8, 15, 16). Using gingival fibroblasts (GFs) derived from ERS patients’ gingiva we also showed that, in mineralization-inducing conditions, GFs were prone to form calcium deposits. Ectopic mineralization was preceded by the significant upregulation of *POSTN*, the transcription factor *RUNX2* and *Alkaline phosphatase* transcripts, all involved in both ectopic mineralization and fibrotic processes (8, 17–19). These observations provided a cellular basis to previous transcriptional analysis involving FAM20A in ECM biomineralization and remodeling (12) and shed some light on the pathogenesis of the gingival phenotype.

Gingival fibromatosis, a pathological gingival overgrowth, is a fibrotic condition that may be caused not only by hereditary factors but also drugs and inflammatory diseases. As such, the hallmark of the disease is pathological deposition of ECM. In drug-induced forms the excessive deposition of ECM was associated with increased levels of TGF-beta (TGFβ) a factor known to be involved in both fibrosis and calcification (20–23). Whether the same pathway is responsible for the gingival phenotype of ERS patients is currently unknown.

GFs are the main cellular constituent of the gingival tissue. GFs deposit ECM and secrete signaling molecules to the surrounding cells that affect the inflammatory response and tissue remodeling. Studying the contribution of the secretome, i.e. the part of proteins secreted by specific cells to the extracellular space, provides essential insights in pathological conditions including fibrosis and calcification (24). The use of conditioned media (CM) can thus lead to the identification of specific protein signatures and pathways that elicit pathological signaling.

We here used CM from human GFs in culture to examine whether the secretome of ERS-derived GFs may contain proteins involved in fibrosis and ectopic calcifications.

We applied a liquid chromatography tandem-mass spectrometry- (LC-MS/MS) based label-free quantitative proteomic approach to differentially analyze the CM of control and ERS-derived GFs cultured in standard conditions. The protein signature of ERS secretomes suggested that the TGFβ signaling pathway could be upregulated in the ERS-derived GFs. This was further confirmed by the activation of phosphor-Smad3 and the overexpression of various targets of the TGFβ pathway, at the mRNA and/or protein level, in both ERS GFs and gingival tissues. TGFβ1 treatment of ERS-derived GFs further amplified the pro-fibrotic/pro-calcific profile indicating that aberrant activation of TGFβ signaling may contribute to the ERS gingival phenotype. In addition to decipher normal and ERS secretomes, our results provide the first molecular link between FAM20A, impaired ECM homeostasis and gingival overgrowth.

## Materials and Methods

### Ethics - Patients Recruitment

Patients were referred for oral rehabilitation at the Reference Center of rare dental diseases (Rothschild Hospital, CRMR O-RARE, Paris, France). Diagnosis of ERS was based on clinical and radiological features (3, 8). Patients and 3 healthy sex- and age-matched controls were recruited following informed consent in accordance with the principles outlined in the declaration of Helsinki. Written informed consent was obtained from probands for the publication of any potentially identifiable images or data included in this article. The samples used were considered as operating waste according to the French law. Samples from probands and controls were harvested during oral rehabilitation and were prepared for histological or cell culture analyses (authorization CODECOH DC-2018-3382).

### Fibroblast Cell Culture

Control and proband gingival fibroblasts were established by plating small pieces of excised gingival on plastic dishes. Cells, particularly gingival fibroblasts, migrate out of the explant and colonize the petri dish. The flasks were filled with low glucose Dulbecco’s modified Eagle’s medium (DMEM) containing 20% fetal calf serum (FCS), 1% non-essential amino acid, penicillin/streptomycin (100mg/mL) and amphotericin B (2 ng/mL). The flasks were then placed in an incubator programmed at 37°C in a humidified atmosphere with 5% CO_2_ and the cell culture medium was changed twice a week until confluence (90% after about 3 weeks). Once at confluence, the gingival fibroblasts (GFs) were trypsinized (Trypsin-EDTA, GIBCO^®^, 1 mL at 0.05%) and single-cell suspensions were seeded in 25 cm^2^ flasks containing low glucose DMEM 10% of FCS, passaged by splitting when they reached confluence, and frozen in liquid nitrogen until use. Cells at passages 3 to 6 were used in all experiments. We checked each cell culture for the morphology and the marker of fibroblasts (fibroblast-specific protein 1 [FSP1]; ab27957; Abcam, Cambridge, UK). We confirmed that cell cultures did not exhibit any morphological changes during the passages, and that FSP1 was clearly detected in these cells (data not shown). Each experiment using these cells was repeated at least three times.

### Secretome Analysis by Mass Spectrometry

A high resolution mass spectrometry (MS)-based approach was used to detect the secreted proteins from GFs cultures. GFs cultures (three controls and four ERS) were seeded and cultured in triplicates, in low glucose DMEM 10% FBS for three days and then in serum-deprived DMEM for two additional days. Each serum free cell supernatant or secretome (Controls, n=9; ERS1, n=3; ERS2, n=3; ERS3, n=3; ERS4, n=2) was analysed in a single-run of LC-MS/MS.

### Sample Preparation for Secretome

Each culture supernatant (serum free) was precipitated with DOC/TCA (0.1%/10%) to obtain a concentrated protein pool. Protein concentration was estimated using Bradford Assay (Biorad). Based on Bradford results, 25µg of proteins of each sample were loaded into a 7% polyacrylamide gel (Acrylamide/Bis-Acrylamide 30% [29:1], Sigma Aldrich) and an electrophoresis was performed (90 minutes at 10-20mA/gel) to stack all proteins in a small piece of gel. After Coomassie blue staining, the revealed protein bands were excised. Proteins were reduced with 5mM dithiothreitol for 40 min followed by alkylation with 20mM iodoacetamide for 40 min in the dark (all products from Sigma Aldrich). After washing steps with water and acetonitrile (Sigma Aldrich), gel bands were submitted to protein digestion by 1µg of trypsin (Promega). After overnight incubation at 37°C, several steps of peptide extraction were performed with of 0.1% formic acid (FA) in water and acetonitrile solutions. Finally, for each sample, peptide fractions were combined and dried.

### Nano LC-MS/MS Analysis

For each sample, peptide fractions were solubilized in FA 0.1% (v/v) and analyzed on a LTQ-Orbitrap Elite apparatus coupled to an Easy nanoLC II system (Thermo Scientific). Peptides were injected onto an enrichment column (C18 Pepmap100, Thermo Scientific). The separation was carried out with an analytical column needle (NTCC-360/100-5-153, Nikkyo-Technos). The flow rate was 300 nL/min and the mobile phase composed of H_2_O/0.1% FA (buffer A) and ACN/0.1% FA (buffer B). The elution gradient duration was 120 minutes: 0-106 min, 2-40% B; 106-110 min, 40-100% B; 110-120 min, 100% B. The mass spectrometer was operated in positive mode with CID fragmentation. For mass spectrometry settings, the capillary voltage was 1.5 kV and the temperature of the capillary was 275°C. The *m*/*z* detection range was 400-1800 in MS scan at a resolution of 60 000. The 20 most intense peptide ions were selected and the fragmentation occurred with a normalized collision energy of 35. Dynamic exclusion of already fragmented precursor ions was applied for 30 seconds.

### Quantification and Statistical Analysis

After MS analysis, raw data were imported in *Progenesis LC-MS* software (NonLinear Dynamics, Newcastle, UK). To perform quantification, one sample was set as a reference and retention times of all other samples were aligned. After alignment and normalization, statistical analysis was performed using the inbuilt Progenesis statistical box called ‘one-way ANOVA’. MS/MS spectra were then exported for peptide identification with Mascot (Matrix Science, version 2.6.0). Database searches were performed with the following parameters: taxonomy: human (22,244 sequences); 1 missed cleavage; variable modification: carbamidomethyl of cysteine and oxidation of methionine. Mass tolerances for precursor and fragment ions were 10 ppm and 0.35 Da respectively. False discovery rates were calculated using a decoy-fusion approach in Mascot. Identified spectrum matches with -10logP value of 20 or higher were kept, at a FDR threshold of 5%. Mascot search results were imported into *Progenesis*. For each condition, the total cumulative abundance of protein was calculated by summing abundances of peptides. Proteins identified with less than 2 peptides were discarded from further analysis.

### Network Biology and Systems Level Analysis of CM Secretome

Secreted proteins identified by MS were entered in STRING database (string-db.org) to create a protein-protein association network (25). Network nodes represent all the proteins produced by a single protein-coding gene locus noting that splice isoforms or post-translational modifications are collapsed. Edges represent protein-protein associations that are specific and meaningful, such as proteins that jointly contribute to a shared function (note that this does not necessarily mean they physically bind each other). Thickness of network edges indicates the strength of data support based on text mining, experiments, databases, co-expression, neighbourhood, gene fusion and co-occurrence. The minimum required interaction score was set to a high confidence level of 0.7. The exported network image was further refined using Cytoscape 3.8.2 (https://cytoscape.org). Significantly deregulated proteins identified in CM secretome were also entered in ToppFun (ToppGene Suite: https://toppgene.cchmc.org/) that detects functional enrichment of the query list based on transcriptome, proteome, regulome (transcription factors and miRNAs) and ontologies (GO, pathway) amongst other features (26). Finally, fold of functional enrichment was determined using the GO resource powered by geneontology.org (PANTHER16.0).

### TGF-β1 Treatment

For RT-qPCR and Western blot analyses cells were seeded at 7000 cells per cm^2^ surface area in 6 well plates and in 100 mm diameter dishes respectively. For immunofluorescence cells chamber slides (Nunc Lab-Tek, Thermofisher) were used. GFs from 3 control and 4 ERS subjects were used for all the experiments. Three replicates of each experiment were performed for each test to ensure reproducibility.

For TGF-β1 treatment, recombinant human TGF-β1 at 5 ng/ml was used (R&D Systems, MN, USA). Prior to TGF-β1 treatment, nearly confluent cells were serum-starved in low-glucose DMEM for 24h and washed with serum-free DMEM. Immediately after, cells were treated with TGF-β1 for 6 hours. Unless otherwise stated ‘ERS GFs’ or ‘mutant CM’ refer to untreated cells/CM.

### Western Blotting

Western blotting was performed as previously described (8). In brief, GFs were washed twice with PBS and protein was isolated in RIPA buffer (Sigma Aldrich) containing protease (Roche Diagnostics) and phosphatase inhibitors (Calbiochem) cocktails. Before loading, protein concentration was determined by Pierce^®^ BCA Protein assay kit (Pierce; Waltham, MA, USA). A 25 µg quantity of protein from each sample was separated by sodium dodecyl sulphate polyacrylamide gel electrophoresis (SDS-PAGE) and transferred to nitrocellulose membranes. Conditioned medium made from GF cultures were directly loaded on the gel. Membranes were washed with Tris-buffered saline containing 0.05% Tween-20 (TBS-T) and blocked with 5% dried milk in TBS-T. Primary antibodies for phosphorylated-SMAD3 (p-SMAD3) (ser423/425) (ab52903; Abcam; 1:1000), Periostin (EPR19934; Abcam; 1:500), FAM20A (OACD03385; Aviva; 1:500), and GAPDH (MAB374; Millipore; 1:2000) were used to incubate the membranes for 12 hours. Detection was with appropriate peroxidase-conjugated secondary antibodies (Jackson ImmunoResearch; West Grove, PA, USA; 1:2000), which were developed with SuperSignal Western Pico Chemiluminescence Substrate (Pierce).

### Quantitative RT-qPCR

Total RNA was isolated using commercially available kits according to manufacturer guidelines (RNeasy Mini, Qiagen) and measured (Nanodrop, Peqleb). One μg was used in a reverse transcription reaction (SuperScript First strand synthesis, Thermofisher). Quantitative-PCR was performed using Quantifast SYBR Green PCR Kit (Qiagen), reactions were performed in triplicate. Transcript levels were calculated using the standard curves generated using serial dilutions of cDNA obtained by reverse transcription of control RNA samples then normalized to HPRT. Primer sequences were listed in **Supplementary Table 1**. Amplification specificities were assessed by melting curve analyses and amplicons were sequenced. Values correspond to the mean of 3 independent experiments in triplicates of three control cultures and the four ERS patient cultures. Data represent mean fold gene expressions ± s.d. relative to control (without TGFβ1). Data were analyzed *via* two-way ANOVA with Bonferroni multiple comparisons test (*p<0.05, **p<0.01, ***p<0.001).

### Immunocytochemistry

*Gingival biopsy*: Approximately, 1 cm^3^ gingival samples from patients were fixed for 24 h at 4°C in 4% paraformaldehyde and then embedded in paraffin wax. After sectioning, epitope retrieval was achieved by heat. Sections were incubated overnight at 4°C with primary antibodies; rabbit anti-Collagen VI (EPR17077; Abcam; 1:250), rabbit anti-phosphoSMAD3 (ab52903; Abcam; 1:100) and mouse anti-Netrin-1 (ALX-522-100; Enzo Life; 1:100).

*GF culture*: Cells were fixed with 4% paraformaldehyde, permeabilized with 0.1% Triton X-100, and blocked with 1% bovine serum albumin. Primary antibodies used were rabbit anti-FAM20A (OACD 03385; Aviva; 1:250) and P-SMAD3 (ab52903; Abcam; 1:100). Golgi staining was achieved by incubating cultures with lectin HPA from *Helix pomatia* Alexa 647-conjugated (Thermo Fisher Scientific; 1:200).

Secondary antibodies used were Alexa 488- or Cy3-conjugated donkey anti-rabbit (Jackson Immunoresearch Laboratories, West Grove, PA; 1:500), and Alexa 488-conjugated donkey anti-mouse (Thermo Fisher Scientific; 1:200). Nuclear staining was achieved by 20 min incubation at room temperature in Hoechst 33342 (Thermo Fisher Scientific). No cellular autofluorescence and no nonspecific labeling were detected in these conditions. Images were collected by confocal microscopy (Zeiss LSM8) and processed using ZEN (Zeiss) and ImageJ softwares. ERS photomicrographs in **Figure 4** are a representative of all ERS cultures.

### Statistical Analysis

Statistical analysis was by one-way or two-way ANOVA, as appropriate, followed by Bonferroni multiple comparisons test with Graphpad Software version 5 (Graphpad Software; La Jolla, CA, USA) (p < 0.05 was considered significant). Data are expressed as the mean ± standard deviation of 3 or 5 individual experiments with independent primary cultures from different subjects. Individual experiments included three replicates.

## Results

### Mass Spectrometry Analysis Overview

Primary cultures from GFs were obtained from three unrelated controls and four ERS patients carrying distinct FAM20A mutations (**Table 1**). We previously showed that the c.358C>T mutation (ERS1) resulted in a null protein, undetectable in ERS1 GFs (8). FAM20A could readily be detected in GFs derived from the other three patients and control subjects (**Supplementary Figure 1**). However, whereas in control GFs FAM20A was essentially localized in discoidal vesicles, most likely secretory ones, in the mutant GFs FAM20A was exclusively detected in HPA positive, cis-Golgi structures (**Supplementary Figures 1A–D**). Western blot analysis of the CM failed to detect secreted FAM20A in either control or ERS GFs (**Supplementary Figure 1E**).

**Table 1:** FAM20A mutations in ERS patients.

Patient	Age (y)	Gender	Exon	Mutation	Effect	References
ERS1	18	Male	Exon 1	c.358C>T	p.G120X	(8)
ERS2	16	Male	Exon 4 Exon 5	c.641_719del79bp c.755_757delTCT	p.I214fsX259 p.F252del	(3)
ERS3	20	Male	Exon 11	c.1432C>T	p.R478X	(3)
ERS4	22	Male	Exon 11	c.1513delA	p.I505fsX506	(3)

Mutations are described on the cDNA and predicted protein changes. Listing of one allele indicated homozygosity; two alleles indicate compound heterozygosity. Every patient had biallelic mutations involving deletions or nonsense changes. Positive family history was found in ERS2 (1 sister) and ERS4 (1sister and 1 brother).

To gain insight into the pathogenesis of gingival fibromatosis we analyzed the proteome of control and ERS-derived CM using nano-LC-MS/MS. In order to evaluate the reproducibility of the experiments, different linear regressions were performed by plotting the logarithm of protein intensities for the different samples of the same group (as mentioned in the Methods section). The averaged regression coefficient measured to evaluate the robustness of the technical scenario between biological replicates for the different groups of samples was estimated as follows, R_Ctls_ = 0.9869, R_ERS1_ = 0.9568, R_ERS2_ = 0.9095, R_ERS3_ = 0.9376, R_ERS4_ = 0.9670. Without any filtering criteria, nano-LC-MS analyses allowed to identify 1061 proteins (**Supplementary Table S2**). After applying classical proteomic filters (at least two unique matched peptides for each protein) this number was decreased to 534 proteins which are further described below. Out of these proteins, 520 were predicted to be secreted (96%), including 187 classically (as based on the presence of a signal peptide using Uniprot; https://www.uniprot.org) and 333 non-classically (**Supplementary Table S3**). The latter also included proteins found in extracellular vesicles (exosomes, ectosomes and apoptotic bodies) as defined in Vesiclepedia (27); (http://microvesicles.org/index.html) and Exocarta (28); (http://exocarta.org/index.html). The 17 remaining hits were most likely representing membrane shed peptides.

The set of the 187 classically secreted proteins were the most abundantly represented in all samples. We used Gene Ontology (GO) analysis to evaluate the molecular functions, biological processes and cellular components, related to these proteins. All *p*-values were adjusted with Bonferroni corrections. The most enriched “GO Molecular Function” represented binding and signalling of ECM structural constituents such as collagens, glycosaminoglycans or integrins (**Table 2**). ECM organization, collagen trimerization, and collagen fibril organization were accordingly the major “GO Biological Process” identified (**Table 2**).

**Table 2:** Most enriched GO terms: molecular function, biological process, cellular component.

Molecular Function	Name	pValue	Bonferroni	Genes from Input	Genes in Annotation
GO:0005201	extracellular matrix structural constituent	7.382E-85	3.071E-82	62	185
GO:0005198	structural molecule activity	4.696E-46	1.954E-43	63	743
GO:0005518	collagen binding	8.911E-31	3.707E-28	24	82
GO:0005539	glycosaminoglycan binding	8.596E-26	3.576E-23	30	252
GO:0005102	signaling receptor binding	5.808E-24	2.416E-21	64	1842
GO:0002020	protease binding	1.035E-22	4.306E-20	31	350
GO:0008233	peptidase activity	2.736E-22	1.138E-19	44	868
GO:0005178	integrin binding	4.904E-22	2.040E-19	23	157
GO:0019838	growth factor binding	2.418E-21	1.006E-18	23	168
GO:0004175	endopeptidase activity	1.300E-20	5.407E-18	37	640
**Biological Process**	**Name**	**pValue**	**Bonferroni**	**Genes from Input**	**Genes in Annotation**
GO:0030198	extracellular matrix organization	3.792E-82	1.601E-78	76	431
GO:0043062	extracellular structure organization	4.581E-82	1.934E-78	76	432
GO:0045229	external encapsulating structure organization	6.673E-82	2.818E-78	76	434
GO:0022610	biological adhesion	1.685E-42	7.115E-39	79	1578
GO:0007155	cell adhesion	1.314E-41	5.549E-38	78	1571
GO:0030199	collagen fibril organization	3.530E-38	1.491E-34	26	63
GO:0001944	vasculature development	1.370E-27	5.785E-24	50	903
GO:0001568	blood vessel development	1.871E-26	7.900E-23	48	866
GO:0001501	skeletal system development	1.963E-26	8.288E-23	41	583
GO:0016477	cell migration	8.552E-25	3.611E-21	64	1812
**Cellular Component**	**Name**	**pValue**	**Bonferroni**	**Genes from Input**	**Genes in Annotation**
GO:0031012	extracellular matrix	5.96E-148	2.14E-145	122	633
GO:0030312	external encapsulating structure	9.09E-148	3.27E-145	122	635
GO:0062023	collagen-containing extracellular matrix	1.505E-147	5.41E-145	115	498
GO:0005788	endoplasmic reticulum lumen	3.938E-54	1.418E-51	53	323
GO:0005604	basement membrane	9.433E-38	3.396E-35	31	122
GO:0005581	collagen trimer	2.900E-25	1.044E-22	21	87
GO:0034774	secretory granule lumen	7.695E-23	2.770E-20	30	324
GO:0060205	cytoplasmic vesicle lumen	1.101E-22	3.965E-20	30	328
GO:0031983	vesicle lumen	1.315E-22	4.734E-20	30	330
GO:0031091	platelet alpha granule	1.522E-21	5.481E-19	19	92

Selection based on p-value and adjusted with Bonferroni correction. Hit count in genome shows the number of genes in a given pathway, and the hit count in query list shows how many genes in the query list are hit in a given GO terms. The full output table generated by ToppFun is shown in **Supplementary Table S4**.

Research of the most enriched pathways (**Table 3**) identified 140 out of 187 secreted proteins as belonging to the “Ensemble of genes encoding extracellular matrix and extracellular matrix-associated proteins”, “Ensemble of genes encoding core extracellular matrix including ECM glycoproteins, collagens and proteoglycans” and “Ensemble of genes encoding ECM-associated proteins including ECM-affiliated proteins, ECM regulators and secreted factors” pathways (**Table 3**).

**Table 3:** Most enriched pathways.

Pathway	Name	Source	pValue	Bonferroni	Genes from Input	Genes in Annotation
M5889	Ensemble of genes encoding extracellular matrix and extracellular matrix-associated proteins	MSigDB C2 BIOCARTA (v7.3)	6.717E-135	6.153E-132	140	1026
M5884	Ensemble of genes encoding core extracellular matrix including ECM glycoproteins, collagens and proteoglycans	MSigDB C2 BIOCARTA (v7.3)	1.567E-85	1.435E-82	75	275
1270244	Extracellular matrix organization	BioSystems: REACTOME	2.416E-69	2.213E-66	67	298
M3008	Genes encoding structural ECM glycoproteins	MSigDB C2 BIOCARTA (v7.3)	1.949E-55	1.785E-52	51	196
M5885	Ensemble of genes encoding ECM-associated proteins including ECM-affiliated proteins, ECM regulators and secreted factors	MSigDB C2 BIOCARTA (v7.3)	1.738E-39	1.592E-36	65	751
M3468	Genes encoding enzymes and their regulators involved in the remodeling of the extracellular matrix	MSigDB C2 BIOCARTA (v7.3)	9.420E-30	8.629E-27	36	238
1270245	Collagen formation	BioSystems: REACTOME	5.373E-29	4.921E-26	26	93
M18	Beta1 integrin cell surface interactions	MSigDB C2 BIOCARTA (v7.3)	2.802E-28	2.566E-25	23	66
M7098	ECM-receptor interaction	MSigDB C2 BIOCARTA (v7.3)	5.997E-24	5.493E-21	22	84
1270256	ECM proteoglycans	BioSystems: REACTOME	4.440E-23	4.067E-20	19	57

Selection based on p-value and adjusted with Bonferroni correction. Hit count in genome shows the number of genes in a given pathway, and the hit count in query list shows how many genes in the query list are hit in a given pathway. The full output table generated by ToppFun is shown in **Supplementary Table S4**.

**Figure 1:**
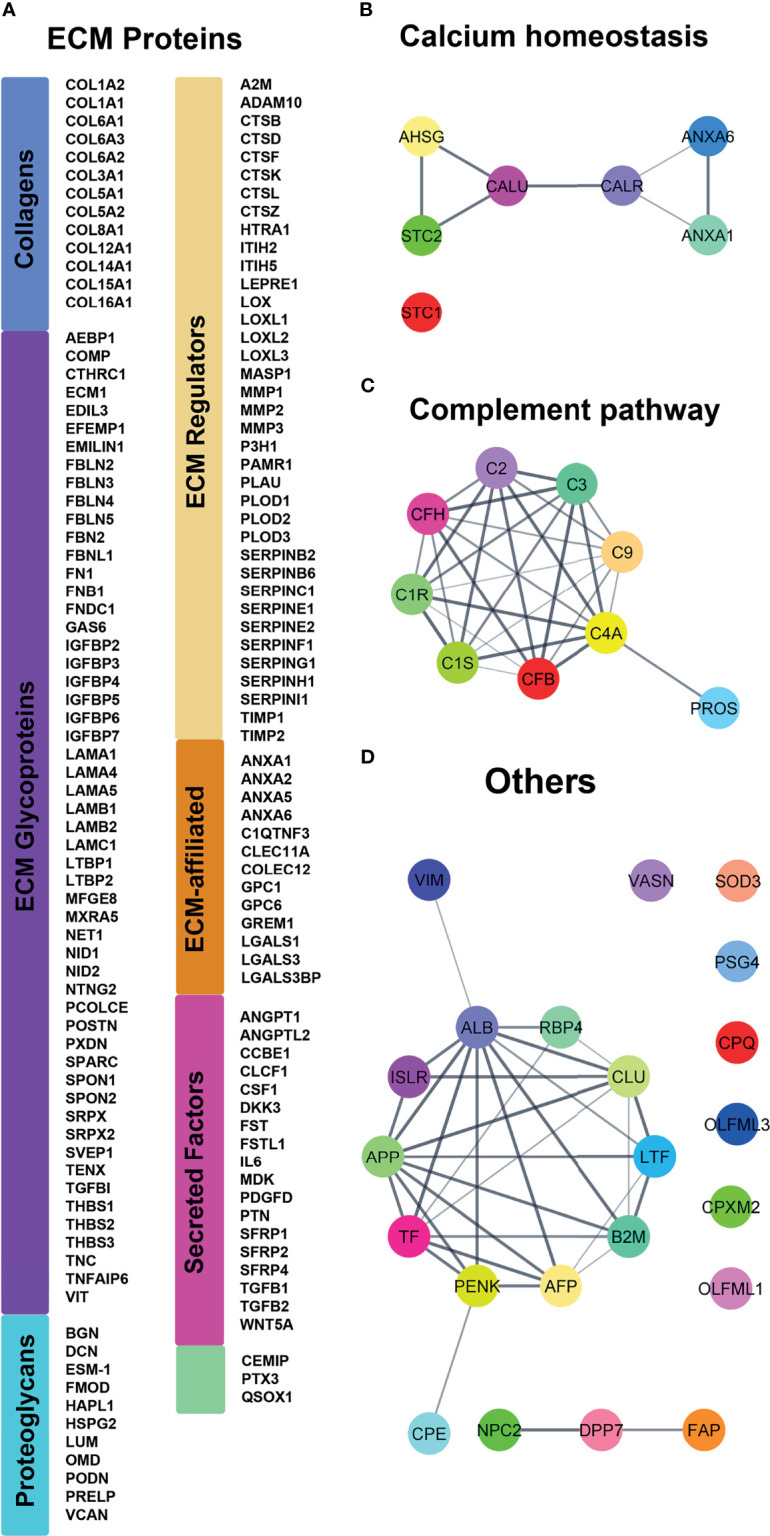
Proteomic analysis of the classically secreted proteins identified in controls and ERS patients secretomes using MS. **(A)** Classification following Naba et al. (29) of the 140 ECM proteins. Proteins are represented by their corresponding gene name (HUGO Gene Nomenclature) for clarity. **(B–D)** String analyses revealed supplementary interactomes of **(B)** calcium interacting proteins, **(C)** complement cascade and **(D)** remaining classically secreted proteins.

In total, 149 out of the 187 proteins were predicted to be linked to structure and organization/remodeling of the ECM (29) (**Figure 1A**) including thirteen different types of Collagens (I, VI, VIII or XII) and several proteoglycans of the small leucine-rich repeat proteins family (Podocan, Biglycan, Decorin, Fibromodulin and Lumican, as well as Versican and the *HSPG2* encoded Perlecan). With the exception of Podocan, all the above small leucine-rich repeat members were previously localized in fibroblasts from human gingiva (30) confirming the accuracy of GFs CM model. Among the other proteins, we identified the ECM regulators Fibronectin, Osteonectin (SPARC), Laminins and BHG3, the collagenolytic enzymes Matrix metallopeptidases I and II, Cathepsins as well as the so-called ‘ECM-affiliated proteins’ Annexins, Galectins, and Glypicans. Furthermore, our analysis pinpointed several secreted growth factors such as Follistatin, Follistatin-like protein 1, Angiopoietin 2 and Transforming growth factor beta 2 (TGFβ2; **Figure 1A**). In addition to the above 149 proteins, we found 7 proteins involved in calcium binding (Calreticulin, Calumenin, Annexins A1 and A6) or calcium homeostasis (Fetuin A, Stanniocalcin 1 and 2). Six of them were forming an interaction network as revealed by String analysis (**Figure 1B**). A second interactome was formed among the 9 proteins of the complement pathway (**Figure 1C**). In addition, 13 out of the 22 remaining secreted proteins were forming a third protein-protein association network (**Figure 1D**), related to lipid metabolism and iron transport and homeostasis.

The abundance of the 333 non-classically secreted proteins was lower; only 7 were relatively well represented (cytoplasmic Actin, Pyruvate Kinase, Prelamin A/C, Glyceraldehyde-3-phosphate dehydrogenase, Filamin-A, 78 kDa glucose-regulated protein, and Endoplasmin).

### Differentially Secreted Proteins Between Control and ERS-1 to -4 Gingival Fibroblasts

**Figure 2:**
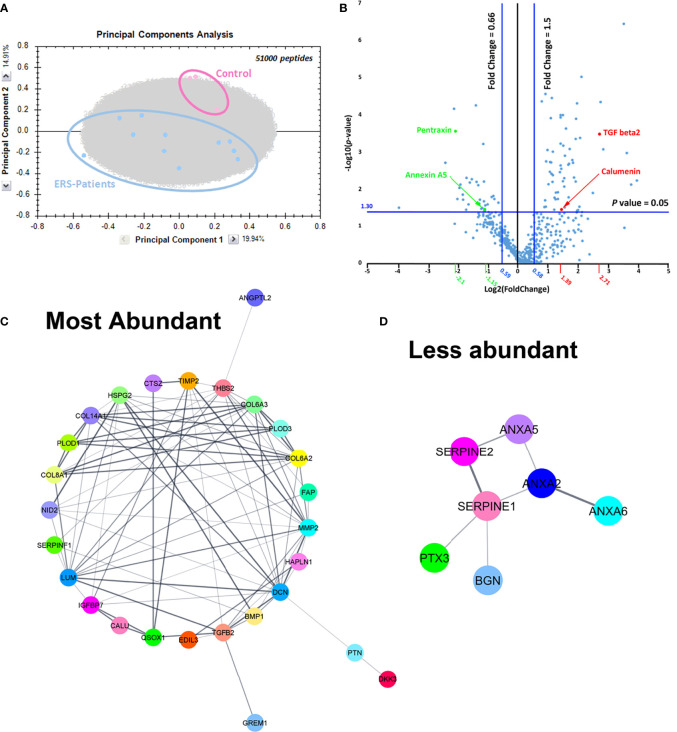
**(A)** Principal component analysis plot including all peptides confidently annotated from MS data, that belong to all proteins identified in controls and ERS patients secretomes. **(B)** Volcano plot representation plotting Log (*p*-value) against Log (fold change). Here, *p*-values were obtained from *t*-test performed for all quantified proteins between Controls and ERS patients. In the right upper quarter, red dots represent examples of proteins that were more abundant in secretomes from ERS patients and green dots represent proteins with lower abundance in secretomes from ERS patients (upper left quarter). **(C, D)** Protein-protein association network using String analysis performed with the differentially secreted proteins, over-expressed **(C)** and under-expressed **(D)** in the ERS secretomes.

The high regression coefficient of the samples analysed allowed a reliable comparison of the control and mutant secretomes. Principal component analysis clearly demonstrated a segregation between control and ERS CM (**Figure 2A**). At the protein level, the volcano plot representation for all the identified proteins revealed a clear separation between more abundant and less abundant proteins (**Figure 2B**). It also allowed to visualize the109 differentially regulated proteins, 69 being more abundant (upper right) and 40 being less abundant (upper left) in CM from ERS compared to controls (**Supplementary Tables S5****,** **S6**). For the purpose of this work, we decided to focus on the 50 classically secreted, differentially expressed proteins; i.e 38 over-represented and 12 under-represented proteins in the mutant CM (**Tables 4**, **5**).

**Table 4:** More abundant classically secreted proteins found in ERS CM.

Accession	Description	Max fold change
CO8A1_HUMAN	Collagen alpha-1(VIII) chain – COL8A1	15.4
HPLN1_HUMAN	Hyaluronan and proteoglycan link protein 1 – HAPLN1	12.2
NET1_HUMAN	Netrin-1 – NET1	11.4
TGFB2_HUMAN	Transforming growth factor beta-2 – TGFB2	6.5
GREM1_HUMAN	Gremlin-1 – GREM1	5.6
MASP1_HUMAN	Mannan-binding lectin serine protease 1 – MASP1	4.4
NPC2_HUMAN	Epididymal secretory protein E1 – NPC2	4.3
PTN_HUMAN	Pleiotrophin – PTN	4.1
ANGL2_HUMAN	Angiopoietin-related protein 2 – ANGPTL2	3.9
SEPR_HUMAN	Prolyl endopeptidase FAP – FAP	3.9
QSOX1_HUMAN	Sulfhydryl oxidase 1 – QSOX1	3.8
DKK3_HUMAN	Dickkopf-related protein 3 – DKK3	3.8
IBP7_HUMAN	Insulin-like growth factor-binding protein 7 – IGFBP7	3.6
TSP2_HUMAN	Thrombospondin-2 – THBS2	3.5
DPP2_HUMAN	Dipeptidyl peptidase 2 – DPP2	3.5
NID2_HUMAN	Nidogen-2 – NID2	3.2
STC1_HUMAN	Stanniocalcin-1 – STC1	3.2
PDGFD_HUMAN	Platelet-derived growth factor D – PDGFD	3.2
SRPX_HUMAN	Sushi repeat-containing protein – SRPX	3.0
TIMP2_HUMAN	Metalloproteinase inhibitor 2 – TIMP2	2.8
MMP2_HUMAN	72 kDa type IV collagenase – MMP2	2.8
SVEP1_HUMAN	Sushi, von Willebrand factor type A, EGF and pentraxin domain-containing protein 1 – SVEP1	2.7
CO6A2_HUMAN	Collagen alpha-2(VI) chain – COL6A2	2.7
CO6A3_HUMAN	Collagen alpha-3(VI) chain – COL6A3	2.7
EDIL3_HUMAN	EGF-like repeat and discoidin I-like domain-containing protein 3 – EDIL3	2.7
CALU_HUMAN	Calumenin – CALU	2.6
COEA1_HUMAN	Collagen alpha-1(XIV) chain – COL14A1	2.5
CEMIP_HUMAN	Cell migration-inducing and hyaluronan-binding protein – CEMIP	2.5
CATZ_HUMAN	Cathepsin Z – CTZ	2.2
FINC_HUMAN	Fibronectin – FN1	2.2
PGBM_HUMAN	Basement membrane-specific heparan sulfate proteoglycan core protein – HSPG2	2.2
CBPQ_HUMAN	Carboxypeptidase Q - CBQ	2.1
LUM_HUMAN	Lumican – LUM	2.1
PGS2_HUMAN	Decorin _ DCN	2.0
PEDF_HUMAN	Pigment epithelium-derived factor – SERPINF1	1.9
PLOD3_HUMAN	Procollagen-lysine,2-oxoglutarate 5-dioxygenase 3 – PLOD3	1.8
BMP1_HUMAN	Bone morphogenetic protein 1 – BMP1	1.7
PLOD1_HUMAN	Procollagen-lysine,2-oxoglutarate 5-dioxygenase 1 – PLOD1	1.5

**Table 5:** Less abundant classically secreted proteins found in ERS CM.

Accession	Description	Max fold change
ESM1_HUMAN	Endothelial cell-specific molecule 1 – ESM1	15.4
PTX3_HUMAN	Pentraxin-related protein - PTX3	4.2
CO9_HUMAN	Complement component - C9	4.0
ISLR_HUMAN	Immunoglobulin superfamily containing leucine-rich repeat protein	3.5
ANXA6_HUMAN	Annexin A6 – ANXA6	3.2
SFRP2_HUMAN	Secreted frizzled-related protein 2 – SFRP2	2.4
LOXL3_HUMAN	Lysyl oxidase homolog 3 – LOXL3	2.4
PAI1_HUMAN	Plasminogen activator inhibitor 1 – SERPINE1	2.2
PGS1_HUMAN	Biglycan – BGN	2.2
ANXA5_HUMAN	Annexin A5 – ANXA5	2.2
GDN_HUMAN	Glia-derived nexin – SERPINE2	1.6
LTBP2_HUMAN	Latent-transforming growth factor beta-binding protein 2 – LTBP2	1.5

### Gene Ontology of Differentially Expressed Proteins

ECM and collagen fibril organization (**Table 6**, highlighted in blue) as well as angiogenesis (**Table 6**, highlighted in yellow) were the most enriched biological processes related to the group of over-expressed proteins (**Table 6** and **Supplementary Table S7**). GO analysis of the molecular functions showed a very strong enrichment in the ECM structural constituents conferring ‘compression resistance’ and ‘tensile strength’. Molecular functions related to collagen, integrin and glycosaminoglycan binding were also significantly enriched (**Table 7** and **Supplementary Table S7**). As expected, in this group of proteins, all the enriched pathways converged to the pathway “Ensemble of genes encoding extracellular matrix and extracellular matrix-associated proteins” (**Supplementary Table S7**; 15 over 21 pathways). GO disease analysis showed that among the five diseases associated to the over-expressed proteins (**Supplementary Table S7**) tumor angiogenesis (*p*-value 2.747E-8) and fibrosis, liver (*p-*value 1.229E-5) were highly significant. In this context, it is interesting to note that the number and size of gingival vessels and fibrotic modifications are typically observed ERS gingival tissue (8, 31).

**Table 6:** Most enriched GO terms: Biological Process, classed hierarchically by fold increase.

GO biological processes	Hit count in query list	Hit count in the genome	Hit in query list	Fold	P value
collagen fibril organization GO:0030199	10	103	PLOD3, TGFB2, COL6A3, PLOD1, COL14A1, COL6A2, COL8A1, LUM, GREM1, BMP1.	60.59	1.23E-11
glycosaminoglycan catabolic process GO:0006027	4	62	HSPG2, LUM, DCN, CEMIP	40.26	3.32E-02
negative regulation of angiogenesis GO:0016525	6	103	HSPG2, THBS2, SERPINF1, TGFB2, PTN, DCN	36.35	1.70E-04
negative regulation of blood vessel morphogenesis GO:2000181	6	104		36.01	1.80E-04
negative regulation of vasculature development GO:1901343	6	105		35.66	1.90E-04
extracellular matrix organization GO:0030198	18	383	HSPG2, TIMP2, PLOD3, TGFB2, COL6A3, HAPLN1, QSOX1, MMP2, NID2, PLOD1, COL14A1, COL6A2, COL8A1, DCN LUM, GREM1, BMP1.	29.33	7.79E-19
extracellular structure organization GO:0043062	18	384		29.25	8.15E-19
external encapsulating structure organization GO:0045229	18	385		29.10	8.92E-19
regulation of angiogenesis GO:0045765	7	282	HSPG2, THBS2, SERPINF1, TGFB2, PTN, DCN, GREM1	15.49	2.81E-03
regulation of vasculature development GO:1901342	7	283	HSPG2, THBS2, SERPINF1, TGFB2, PTN, DCN, GREM1	15.27	3.08E-03
angiogenesis GO:0001525	7	318	HSPG2, MMP2, COL8A1, GREM1, FAP, ANGPTL2, FN1	13.74	6.19E-03
blood vessel morphogenesis GO:0048514	8	492	HSPG2, MMP2, COL8A1, GREM1, FAP, ANGPTL2, FN1, TGFB2	12.18	2.16E-13

Selection based on p-value and adjusted with Bonferroni correction. Hit count in genome shows the number of genes in a given pathway, and the hit count in query list shows how many genes in the query list are hit in a given GO term. In BLUE, themes belonging to ECM organization along with collagen fibril organization and glycosaminoglycan catabolic processes and in YELLOW terms belonging to angiogenesis. The full output table generated by ToppFun is shown in **Supplementary Table S7**.

**Table 7:** Most enriched GO terms: Molecular function, classed by fold increase.

GO molecular function	Hit count in query list	Hit count in the genome	Hit in query list	Fold	P value
extracellular matrix structural constituent conferring compression resistance GO:0030021	4	22	HSPG2, HAPLN1, LUM, DCN.	>100	2.32E-4
extracellular matrix structural constituent conferring tensile strength GO:0030020	4	41	COL6A3, COL6A2, COL14A1, COL8A1.	60.89	2.27E-3
collagen binding GO:0005518	6	70	COL14A1, LUM, COL6A2, DCN, NID2, FN1	53.49	6.15E-6
extracellular matrix structural constituent GO:0005201	14	173	HSPG2, THBS2, SRPX, HAPLN1, COL8A1, COL6A2, COL6A3, LUM, COL14A1, EDIL3, IGFBP7, NID2, DCN, FN1.	50.50	3.20E-17
integrin binding GO:0005178	6	147	HSPG2, TIMP2, PTN, FAP, EDIL3, FN1.	25.47	4.16E-4
glycosaminoglycan binding GO:0005539	6	234	THBS2, HAPLN1, PTN, DCN, FN1, CEMIP.	16.00	5.83E-3

Selection based on p-value and adjusted with Bonferroni correction. Hit count in genome shows the number of genes in a given pathway, and the hit count in query list shows how many genes in the query list are hit in a given GO term. The full output table generated by ToppFun is shown in **Supplementary Table S7**.

GO analysis of the 12 under-expressed proteins showed an enrichment in ‘regulation of coagulation’, ‘ECM organization’, and ‘regulation of wound healing’ biological processes (**Supplementary Table S8**). Enzymatic regulation by (endo)peptidase activity (Serpine1, Serpine2 and ESM1) and calcium binding (ANXA2, ANXA5 and ANXA6) were the most significant molecular functions. Not surprisingly the most enriched pathways were “Ensemble of genes encoding extracellular matrix and extracellular matrix-associated proteins “and “Dissolution of fibrin clot” (**Supplementary Table S8**). Tumor angiogenesis (*p*-value 8.847E-5) and idiopathic pulmonary fibrosis (*p*-value 2.188E-3) were significantly associated diseases (**Supplementary Table S8**).

Moreover, 33 out of the 38 over-expressed proteins were structural (HAPLN1, Lumican, Decorin, Perlecan and Collagens type 8, type 6 and type 14) or regulating/remodeling factors (Fibronectin, BMP1, Thrombospondin 2, Nidogen 2, IGFBP7, EDIL3, QSOX1, FAP, SerpinF1, PLOD3, Cathepsin Z, TIMP2, MMP2 and Gremlin 1). STRING analysis revealed interactions among all these proteins (**Figure 2C**).

Ten out of the 12 under-represented proteins were ECM-associated proteins (**Table 5**). This set comprised the membrane traffic proteins, Annexin 2 (ANXA2) and 5 (ANXA5) involved in calcification and fibrosis (32–34), the serine protease inhibitors Serpine1 and Serpine2, Pentraxin 3 (PTX3) and the SLRP family member Biglycan (BGN) involved in ECM organization. Interactions between ANXA2, ANXA5, ANXA6, Serpine1, Serpine2, BGN, PTX3 are shown in **Figure 2D**.

### Gene Expression of Differentially Secreted Proteins and Effect of TGF Beta

**Figure 3:**
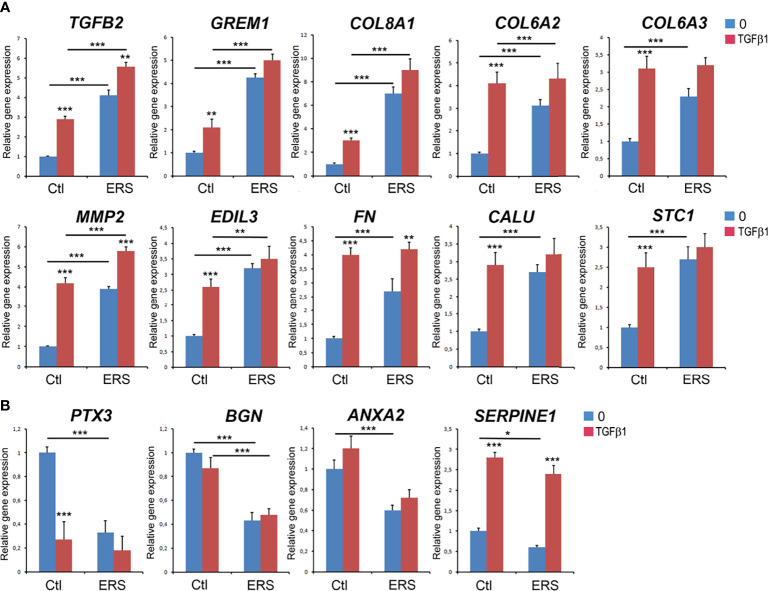
Real Time RT-PCR analysis of candidate genes corresponding to secreted proteins with differential abundance characterized in proteomic analysis. GFs from control and ERS cultured without TGFβ1 (blue columns) or with TGFβ1 (5 ng/ml; red columns) for 6 hours. **(A)** Selected secreted candidates with increased abundance: *TGFβ2*, *Gremlin 1* (*GREM1*), *Collagen alpha* (1) *type VIII* (*COL8A1*), *Collagen alpha* (2) *type VI* (*COL6A2*), *Collagen alpha* (3) *type VI* (*COL6A3*), *Matrix Metallopeptidase 2* (*MMP2*), *EGF Like Repeats and Discoidin Domains 3* (*EDIL3*), *Fibronectin* (*FN*), *Calumenin* (*CALU*) and *Stanniocalcin 1* (STC1). **(B)** Selected secreted candidates with decreased abundance: *Pentraxin 3* (*PTX3*), *Biglycan* (*BGN*), *Annexin A2* (*ANXA2*), and *Plasminogen Activator Inhibitor-1* (*Serpine1*). Control values correspond to the mean of 3 independent experiments in triplicates of three control cultures. ERS values correspond to the mean of 3 independent experiments in triplicates of the four ERS patient cultures. Datas represent mean fold gene expressions ± s.d. relative to control (without TGFβ1). Data was analyzed *via* two-way ANOVA with Bonferroni multiple comparisons test (*p < 0.05, **p < 0.01, ***p < 0.001).

We used lysates of GFs cultured under standard conditions to analyse the mRNAs levels of ten significantly over-represented proteins (*TGFB2*, *Gremlin 1*, *Collagen alpha* (1) *type VIII*, *Collagen alpha* (2) *type VI*, *Collagen alpha* (3) *type VI*, *Matrix Metallopeptidase 2*, *EGF Like Repeats and Discoidin Domains 3*, *Fibronectin*, *Calumenin* and *Stanniocalcin 1*) and of four significantly under-represented ones: *PTX3*, *BGN*, *ANXA2* and *Serpine1* (blue columns in **Figures 3A, B**). Increased mRNA levels were identified for all the over-represented proteins, suggesting that increased protein synthesis may at least partly explain their abundance in the mutant CM (**Figure 3A**, blue columns). Similarly, the transcripts encoding the selected under-represented proteins were significantly downregulated, with a dramatic decrease in *PTX3* mRNA level (5-fold; **Figure 3B**, blue columns).

TGFB2, a multi-functional TGFB isoform with pro-fibrotic and pro-calcific functions was significantly increased in the mutant CM and cell lysates at the protein and mRNA levels respectively (**Table 4** and **Figure 3A**, blue column). The *TGFB2* gene was recently identified as a key factor in drug-induced gingival overgrowth and autocrine TGFβ2 signaling could contribute to the pathogenesis of hereditary or pharmacological-induced gingival fibromatosis (23, 35–37). Additionally, TGFβ2 was shown to favor ectopic calcification in various cell types including vascular smooth muscle cells, dermal fibroblasts and trabecular meshwork cells (38, 39). To exert these effects TGFβ2 employed the canonical Smad-signaling pathway, a pathway also shown to induce Gremlin1 or COL8A1 expression in various cell types (39–43).

We therefore hypothesized that impaired TGFβ signaling could contribute to the dysregulation of the ERS secretome and the pathogenesis of the ERS gingival phenotype.

In canonical TGFβ signaling binding of TGFβ1 or TGFβ2 to and activation of TGFβ- receptors results in the phosphorylation of the intracellular effectors, the cytoplasmic SMAD2 and SMAD3 proteins. The ‘activated’ phosphorylated SMAD2/3 complex translocates to the nucleus and modulates the expression of genes regulated by TGFβ (44).

To further investigate whether TGFβ signalling was intrinsically activated we analysed control and mutant GFs treated or not with recombinant TGFB1 (5ng/ml, 6h). In control GFs, TGFβ1 exposure significantly upregulated the transcription level of the selected genes; a dramatic increase (4-fold) was observed for *MMP2* and *Fibronectin* (*FN1*) (**Figure 3A**, red columns). It is interesting to note that the levels of *MMP2*, *Col6A2*, *Col6A3*, *FN1*, *EDIL3*, *Calumenin* and *Staniocalcin1* mRNA in treated control cells were similar to those of untreated ERS cells (**Figure 3A**, compare red Ctl to blue ERS columns). Exposure of the ERS GFs to TGFβ1 further and significantly increased the expression of *TGFβ2*, *MMP2* and *FN1* mRNA (**Figure 3A**). This observation may suggest that aberrant autocrine activation of the TGFβ pathway could contribute to the gingival phenotype of ERS patients.

The TGFβ1 treatment had contrasting effects on the gene expression of under-represented proteins (**Figure 3B**). Compared to the untreated controls, *PTX3* mRNA level was dramatically decreased; *Serpine1* mRNA level was significantly increased while *BGN* and *ANXA2* mRNA levels were unchanged after treatment (**Figure 3B**, blue and red Ctl columns). Exposure of ERS cultures to TGFβ1 did not change the levels of *PTX3*, *BGN* or *ANXA2* mRNA. We only observed an increase in *Serpine1* mRNA levels (**Figure 3B**). It is interesting to note however that the mRNA levels of *PTX3* and *Serpine1* were very similar in treated control and ERS-derived GFs (**Figure 3B**, red Ctl and ERS columns). This set of results furthers supports the role of TGFβ and may reflect the contribution of additional pathways.

**Figure 4:**
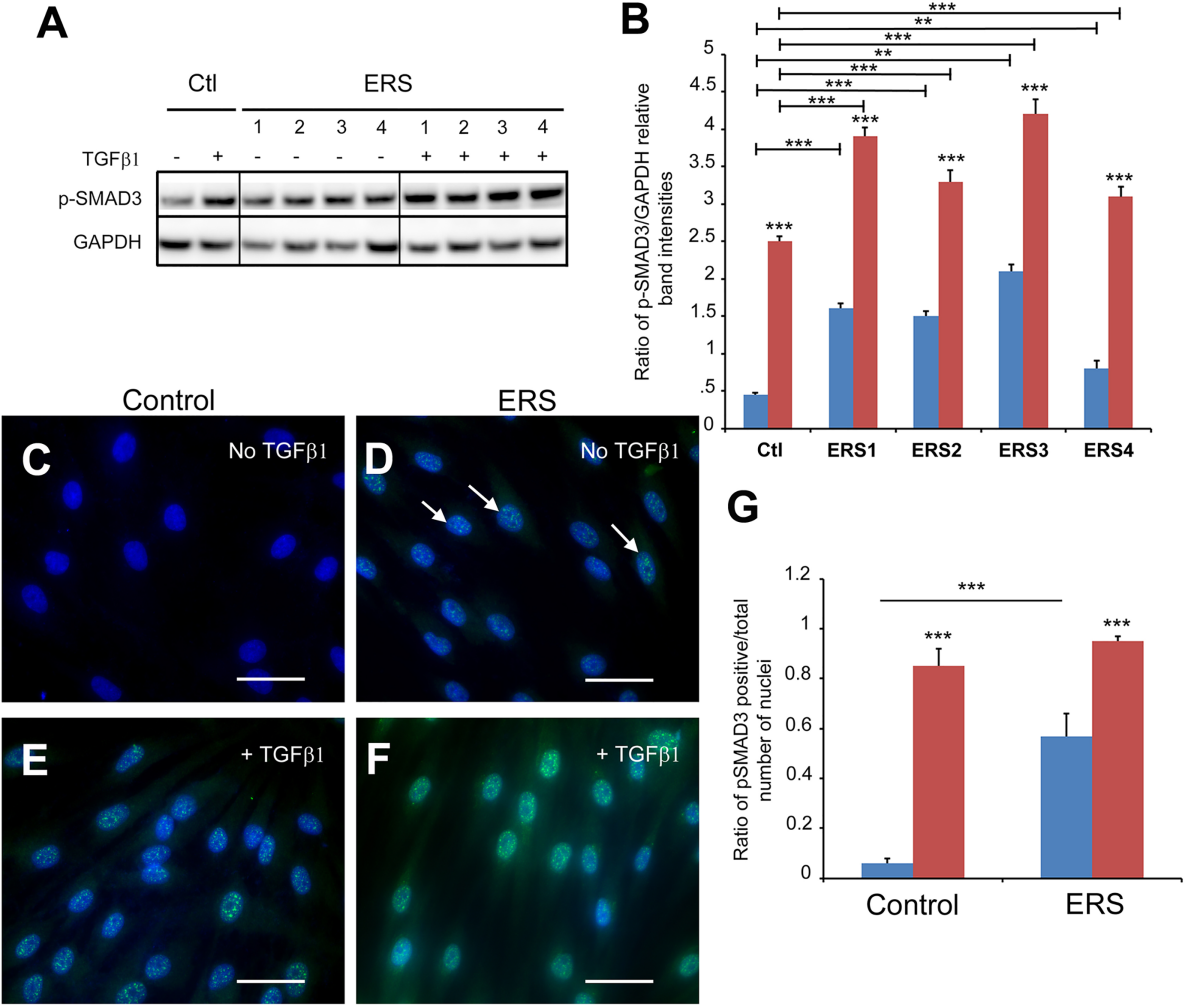
SMAD3 activation in untreated ERS GFs and treated control and ERS GFs. GFs from control and ERS cultured without TGFβ1 (blue columns) or with TGFβ1 (5 ng/ml; red columns) for 6 hours. **(A)** Western blots were performed on cell lysates. P-SMAD3 protein levels were increased in control GFs cultured with TGFβ1. P-SMAD3 protein levels were increased in ERS GFs cultured without or with TGFβ1 compared to Control. **(B)** Densitometric analysis of Phospho-SMAD3 bands normalized to corresponding GAPDH bands. Data represent mean fold change in band intensity ± s.d. relative to GAPDH of 3 independent experiments in triplicates. Data was analyzed *via* two-way ANOVA with Bonferroni multiple comparisons test (**p < 0.01, ***p < 0.001). **(C–F)** Immunocytochemical staining of control **(C, E)** and ERS **(D, F)** GFs cultured without TGFβ1 **(C, D)** or with TGFβ1 (5 ng/mL) **(E, F)** for 6 hours. Cells were fluorescently labeled for p-SMAD3 (green) and nuclei (blue). Co-localization of p-SMAD3 and nuclei indicate nuclear translocation of p-SMAD3. ERS photomicrographs is a representative of all ERS cultures. **(G)** Average ratios of p-SMAD3-positive GFs normalized to total number of cells per field of view at 40X magnification were quantified from 20 images per condition. Data represent mean ratio ± s.d. of 3 independent experiments in triplicates of three control GF cultures and the four ERS patient cultures. Data were analyzed using two-way ANOVA with Bonferroni multiple comparisons test (***p < 0.001). Scale bars: 50 μm.

In agreement with the proteomic and quantitative RT-PCR data suggesting activation of the TGFβ pathway the levels of the effector protein phospho-SMAD3 (p-SMAD3) was significantly increased in treated control (**Figures 4A, B** red column) as well as treated and untreated ERS GFs (**Figures 4A, B** blue and red ERS columns). Immunomorphological data showing nuclear accumulation of p-SMAD3 in untreated ERS GFs (**Figure 4D**) and in treated control and ERS GFs (**Figures 4E, F**) further suggested that this pathway was intrinsically activated in ERS GFs. The ratio p-SMAD3-positive nuclei/total number of nuclei was significantly increased in untreated ERS GFs compared to untreated control GFs (**Figure 4G**, blue columns). Upon TGFβ1 exposure a tenfold increase in the ratio of p-SMAD3-positive nuclei to the total number of nuclei in control GFs was seen (**Figures 4G**, Ctl). In treated ERS GFs, a further increase in this ratio was seen but it was not significantly different from that of treated control GFs (**Figure 4G**).

**Figure 5:**
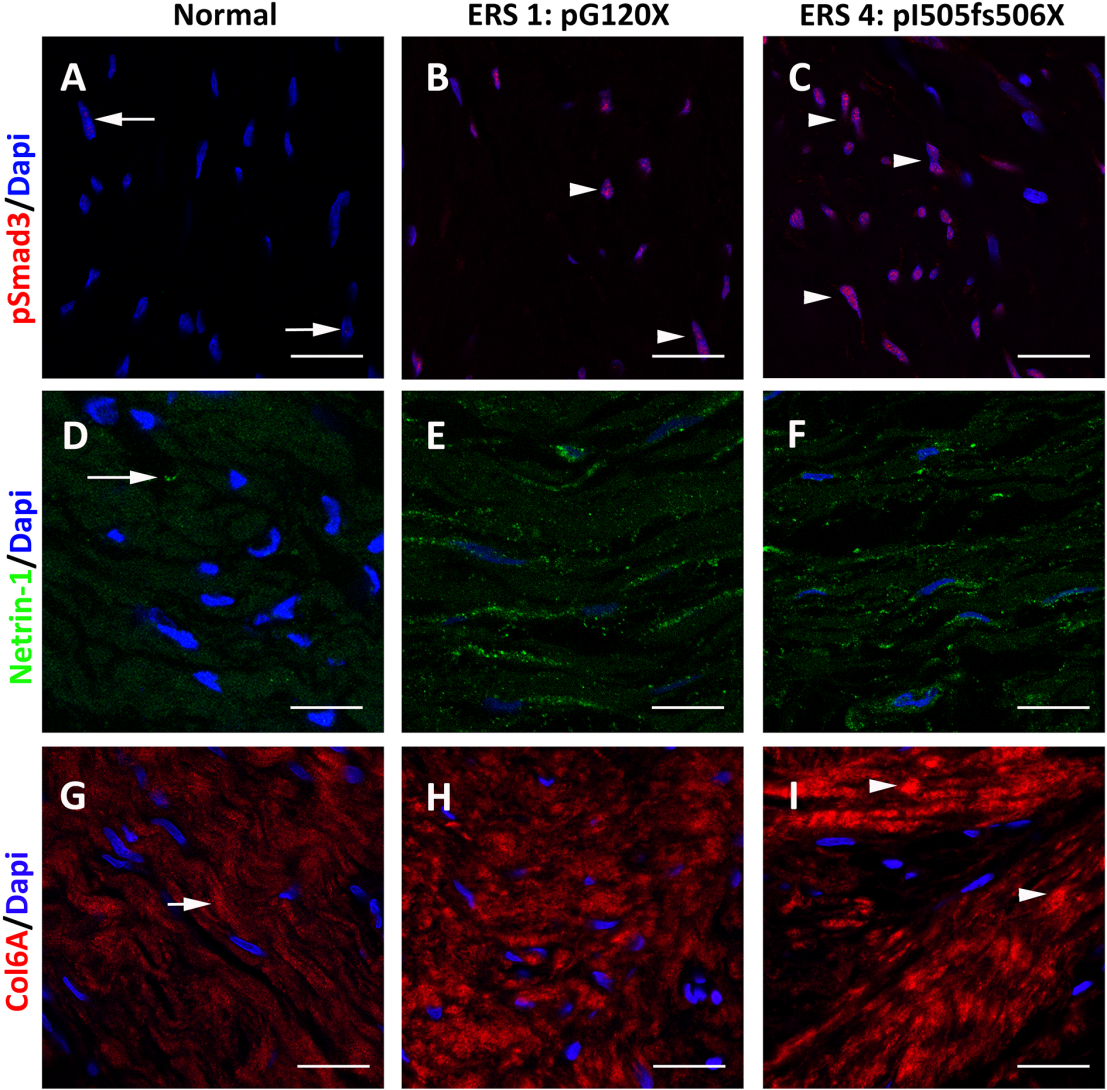
Representative p-SMAD3 staining in gingival tissues from healthy subject **(A)** and ERS patients **(B, C)**. **(A)** A small number of nuclei (arrows) displayed p-SMAD3 immunoreactivity in control gingiva. **(B, C)** The number of p-SMAD3 immunoreactive nuclei (arrowheads) was dramatically increased in ERS patients. **(D)** Netrin1 was expressed in discrete spots at fibroblast extremities. **(E, F)** In ERS patients, Netrin1 immunoreactivty was found in intense spots along the entire fibroblast soma. **(G)** Fibrous Collagen VI immunoreactivity was found in the Ctl connective tissue (arrow). **(H, I)** In ERS patients, collagen VI immunoreactivity was increased and expressed along thick and disorganized fibers. Scale bars: **(A–C)**, **(G–I)**: 50 μm; **(D–F)**: 20 μm.

To determine whether the results obtained *in vitro* reflected what occurs in the ERS gingiva we analysed the distribution of p-Smad3 and the TGFβ targets Netrin1 and COL6A in the gingival connective tissue of unaffected subjects and ERS patients (**Figure 5** and **Supplementary Figure 2**). P-Smad3 was only occasionally seen in control gingiva (**Figure 5A** and **Supplementary Figure 2A**) but was readily observed within the nuclei of ERS fibroblasts (**Figures 5B, C** and **Supplementary Figures 2B, C**). Netrin1, a laminin-like protein involved in angiogenesis, tumor progression and fibrosis (45, 46) exhibited a scattered distribution within fibroblasts of the control gingiva (**Figure 5D**). In agreement with the mass spectrometry data the Netrin-1 staining was much stronger in the ERS gingiva and Netrin1 positive puncta could be seen along the entire ERS fibroblasts (**Figures 5E, F**). Netrin1 has previously been involved in the intercellular cross-talk among bone cells (47); its strong expression may reflect the osteogenic potential of ERS GFs (8). Finally, the expression of the fibrillar COL6A, low in the control gingiva, was patchy and strongly decorated the disorganized collagen fibres of the ERS gingiva (**Figures 5G–I** and **Supplementary Figures 2D–F**). This set of results clearly supports the hypothesis that aberrant activation of the TGFβ pathway may contribute to the ERS gingival phenotype.

## Discussion

We here differentially analyzed the secretomes from control human GFs and GFs carrying 4 distinct *FAM20A* mutations. We identified an ERS-specific protein signature composed of 109 dysregulated proteins including the overexpressed COL8A1, HAPLN1, Netrin 1, TGFβ2 and Gremlin 1 as well as the under-represented PTX3, C9, BGN, Serpine1 and Serpine2, ANXA2 and ANXA5.

We correlated the proteomic data of dysregulated proteins with the respective transcript levels in cell lysates. We found a similar trend for almost all the proteins analyzed indicating that the dysfunction of FAM20A in GFs primarily affected protein synthesis. Since most of the overexpressed proteins are known to be directly or indirectly regulated by TGFβ signaling, an important actor of ECM deposition and remodeling (48) we further investigated whether effectors and targets of this pathway were modified in the ERS gingival tissue. The *in vivo* overexpression of Netrin-1 and COL6A as well as the nuclear accumulation of p-Smad-3 indeed supported the idea that aberrant TGFβ signaling may contribute in the pathogenesis of ERS gingival fibromatosis.

Consistent with the ERS gingival phenotype, characterized by ECM accumulation and calcium deposition, most of the dysregulated proteins have biomineralization functions (49) and were previously associated to ectopic calcification and/or fibrosis (21, 50–53).

In particular, COL8A1, a network forming collagen necessary for migration and proliferation of vascular cells (54) was more than 15-fold increased in the mutant CMs. Increased COL8A1 levels are observed during the development of atherosclerosis or after injury and are known to favor cancer progression (55). *COL8A1* mRNA is induced by TGFβ1 and has been shown to stimulate *MMP2* synthesis in smooth muscle cells during vascular remodeling (56, 57). In ERS GFs, MMP2 protein and *MMP2* mRNA level were 3-fold increased both at the protein (CM) and transcript levels (cell lysates) suggesting that increased expression of COL8A1 and MMP2 would contribute to the pathogenesis of gingival fibromatosis. Supporting this hypothesis GFs exposure to TGFβ1 further increased the levels of *MMP2* transcripts.

That was also the case for COL6A2 and COL6A3 that are fibrillar collagens playing an important role in ECM organization. Both are involved in the pathogenesis of various myopathies and their upregulation, including by TGFβ signaling is associated with many cancer types or fibrosis (58). The abundance of COL6A2 and COL6A3 was much higher in the mutant CM in agreement with the increased mRNA levels and the immunostaining data. A further transcript upregulation was observed after exposure to TGFβ1 arguing that, as in adipose tissue fibrosis (59), a positive TGFβ/COL6 feedback loop may operate in the ERS gingiva to induce expression of additional collagens. It is very interesting to note that the concomitant upregulation of *COL8A1*, *COL6A2* and *COL6A3* has been identified as part of the *adamantinomatous craniopharyngioma* signature (60), an aggressive rare pediatric brain tumor in which calcifications are a diagnostic hallmark.

Besides, HAPLN1, a cross-linking protein that stabilizes the interactions between hyaluronan and chondroitin sulfate proteoglycans or Aggrecan was strongly overexpressed (>12-fold) in the mutant CM. HAPLN1 has been shown to stimulate aggrecan synthesis in the cartilage (61) and changes in HAPLN1 expression could impact collagen organization. HAPLN1 induced by TGFβ in lung fibroblasts is thought to be involved in lung fibrosis (62). Furthermore, increased expression of Aggrecan has also been associated with vascular calcification (63, 64). We previously showed that in ERS gingiva, Aggrecan was aberrantly expressed and mainly localized within/around mineral deposits (8). The concurrent overexpression of the HAPLN1 and aggrecan is compatible with the ERS gingival phenotype and so is strong and concurrent overexpression of TGFβ2 and Gremlin1, possibly involving these factors in ‘feed-forward’ pro-fibrotic and pro-calcific pathway. Alternatively, a high expression of *Gremlin 1* could be protective during early steps of gingival fibromatosis whereas a progressive downregulation could contribute to formation of calcium deposits. It is interesting to note that *Gremlin 1* levels are differentially regulated in some pathological conditions including coronary artery disease (65).

Among the other over-expressed proteins several ones, including Decorin, Lumican, Thrombospondin, Fibronectin and MMPs, have been clearly associated with bone formation and remodeling as mineral matrix formers, nucleation assisters or remodelers (49). Additionally, the calcium binding proteins Calumenin, EDIL3 and Stanniocalcin 1 associated with the initiation of mineralization were more than 2-times increased in the mutant CM, observations compatible with the calcifying potential of ERS GFs (8) and the literature data on these proteins.

Calumenin, a six-EF-hand calcium-binding protein, can be secreted out of the cell and may act in an autocrine manner to modulate rearrangement of cytoskeletal proteins (66). Overexpression of Calumenin is thought to favor ECM mineralization and has been associated with vascular calcification (67). EDIL3 is an ECM protein that acts as a pro-angiogenic and anti-inflammatory factor. EDIL3 was shown to activate TGFβ signaling (68) and its capacity to bind calcium ions and extracellular vesicles (69) suggests a role in calcification.

Stanniocalcin 1 is a glycoprotein that acts in a paracrine and autocrine fashion to maintain phosphate and calcium metabolism and is usually overexpressed in tumoral tissues and during lung fibrosis (70). The upregulation of Stanniocalcin 1 is induced by TGFβ1 and is thought to protect the damaged tissues by maintaining local homeostasis. Whether a similar protective role can be attributed to Stanniocalcin 1 in the pathological ERS gingiva requires further investigation.

We and others previously documented the low inflammatory status of the ERS gingiva (8, 31). The low abundance of the pro-inflammatory molecule PTX3, an acute phase protein increased during aggressive periodontitis (71), or the complement component C9 is in agreement with the histological and clinical findings reported. Furthermore, lower PTX3 levels were associated with liver fibrosis progression (72) whereas in mouse models PTX3 deficiency was associated with excessive fibrin accumulation, augmented collagen deposition and defective tissue repair (73). It is thus possible that low PTX3 amounts may influence progression of both gingival fibromatosis and inflammation.

It was also previously reported that Annexins, calcium-binding proteins with anti-inflammatory, wound healing and defense responses were normal constituents of healthy gingiva and the gingival crevicular fluid. Higher levels of Annexins in these tissues may be associated with a healthy periodontal status (74–76). ANXA2 and ANXA5 were both under-expressed in the mutant GFs; we anticipate that dysregulated responses of ERS GFs to chronic inflammatory and fibrotic conditions may contribute to the fibromatosis. Serpine1, the Plasminogen activator inhibitor 1, is often highly expressed in fibrotic tissues where it favors accumulation of Fibrin and other ECM components (77). Increased Serpine1 expression was indeed shown to play a role in the cyclosporine-induced gingival overgrowth (78). In this model however *Serpine 1* could be independently induced by HIF1α (79). *Serpine1* deficiency was reported to promote spontaneous cardiac fibrosis and in these patients plasma TGFβ levels were upregulated (80). In our hands, the short TGFβ treatment upregulated *Serpine1* in both control and mutant GFs. SERPINE 1 was however 2 times decreased in the mutant GFs. This observation may suggest that the upregulation of *Serpine 1* is an early event not sustained in time.

The cytokine SFRP2, a soluble inhibitor of the canonical Wnt pathway, known to suppress osteoblast differentiation and bone mineralization (81) was decreased in the mutant GFs. It is interesting to note that DKK3, a Wnt modulator and positive target of TGFβ1 signaling (82) was 3.8 times overexpressed suggesting that the TGFβ-Wnt signaling cross-talk was perturbed in the mutant GFs.

It is noteworthy to mention that the matricellular protein connective tissue growth factor (CCN2) a TGFβ target, previously detected by transcriptomic analysis, albeit in low levels, in the gingival tissue from one ERS patient (12) has not been identified in any one of the 4 ERS secretomes analyzed here. Although CCN2 expression in the ERS gingiva has never been published, it is possible that, like previously reported in HGF by Kantarci et al. (83), the extracellular (secreted) protein levels of CCN2 are much lower than the intracellular ones.

In that case the lack of CCN2 protein may be due to the experimental design: conditioned media were collected after a 48h period of serum deprivation (longer periods of serum deprivation may be deleterious for GFs and are avoided prior to secretome analyses). It is thus possible that the amount of CCN2 secreted in 48h is below the detection limit of our LC-MS/MS analysis.

Very little is currently known about the physiological FAM20A distribution and activities. Endogenous FAM20A expression has only been investigated in murine dental/skeletal cells and embryonic fibroblasts (1, 4). In these studies, FAM20A was exclusively found in cell lysates.

Our results agree with the above observations and indicate that in human gingival fibroblasts FAM20A is located within intracellular compartments. The staining was vesicular most likely representing FAM20A along the secretory pathway, albeit not in the cis-Golgi. We did not see FAM20A in the CM of human gingival fibroblasts using either western blot or mass spectrometry analyses; we cannot however exclude that minute amounts of FAM20A may be secreted. It was beyond the scope of this work to detail FAM20A expression; we however clearly showed that the mutations analyzed profoundly modified the intracellular distribution of FAM20A. ERS1 resulted in a null protein and was previously described (8). ERS3 and 4 resulted in C-terminal truncated proteins that were abnormally located within the cis-Golgi. It is interesting to note that the ERS2 mutation, p.F252del, is located within the critical interface, necessary for FAM20A homodimerization (84). ERS2 may not affect FAM20A stability, as previously reported for other mutations within the same interface (84). Indeed, a strong FAM20A signal was observed in the mutant gingival fibroblasts but was abnormally localized in the cis-Golgi. This specific mutation has never been analyzed but seems to be sufficient to alter the subcellular distribution of FAM20A.

According to the available data (84) ERS2 would alter the capacity of FAM20A to act as a FAM20C activator. Nevertheless, the same would stand for ERS1, 2, 3 and 4 (8) as previously reported for FAM20A mutations associated with amelogenesis imperfecta (13).

Whether and how the above mutations modify FAM20C activity in the gingiva are open questions. It is interesting to note however that lack or ectopic expression of FAM20A similarly activate the TGFβ pathway. This novel finding extends our past data (8) and suggests that the aberrant ECM modification and the osteogenic-like transformation of ERS GFs are at least partly supported by aberrant autocrine TGFβ signaling.

In sum, our data provided the first secretomic analysis of ERS gingival fibroblasts and uncovered the hitherto unknown involvement of the TGFβ signaling cascade in ERS gingival fibromatosis. We propose that as previously described for other diseases (85, 86), the dysfunction/mislocalization of FAM20A may favor the abnormal secretion of selected mineral-interacting, extracellular proteins and create a specific microenvironment that facilitates the progression of the gingival disease.

## Data Availability Statement

The datasets presented in this study can be found in online repositories. The names of the repository/repositories and accession number(s) can be found here: ProteomeXChange PXD028003.

## Ethics Statement

Ethical review and approval was not required for the study on human participants in accordance with the local legislation and institutional requirements. The patients/participants provided their written informed consent to participate in this study.

## Author Contributions

OC, PC, and RK conceived the study. AB, CC, and AA participated in the design of the study. VS, CG, OC, PC, and RK wrote the manuscript. VS, CG, LA, LD, CR, and VC performed the experiments. VS, CG, and LA analyzed the data. MB participated in histological study. All authors contributed to the article and approved the submitted version.

## Funding

This study was supported by INSERM and Idex SPC “Once upon a tooth” (grant number: ANR-11-IDEX-0005-02; AB), FHU-DDS ParisNet (Université de Paris, AP-HP, INSERM), ECOS-Nord (C21501) and CAPES/COFECUB (#918/2018). This study contributes to the IdEx Université de Paris ANR-18-IDEX-0001.

## Conflict of Interest

The authors declare that the research was conducted in the absence of any commercial or financial relationships that could be construed as a potential conflict of interest.

## Publisher’s Note

All claims expressed in this article are solely those of the authors and do not necessarily represent those of their affiliated organizations, or those of the publisher, the editors and the reviewers. Any product that may be evaluated in this article, or claim that may be made by its manufacturer, is not guaranteed or endorsed by the publisher.
