# Supplementary material for: Pathogenesis of Enamel-Renal Syndrome Associated Gingival Fibromatosis: A Proteomic Approach
Source: Front Endocrinol (Lausanne). 2021 Oct 29;12:752568. doi: 10.3389/fendo.2021.752568 (PMC8586505; doi:10.3389/fendo.2021.752568)
Supplement: Supplementary file 1 [file DataSheet_1.docx]

**Supplemental Informations to**

**Pathogenesis of enamel-renal syndrome associated gingival fibromatosis: a proteomic approach**

Victor SimancasEscorcia^1^†, Clément Guillou^2,3^†, Lilia Abbad^4^†, Louise Derrien^1^, Claudio Rodrigues Rezende Costa^5^†, Vidjea Cannaya^1^, Mourad Benassarou^6^, Christos Chatziantoniou^4^, Ariane Berdal^1,7^, Ana Carolina Acevedo^5^, Olivier Cases^1^‡, Pascal Cosette^2,3^‡, Renata Kozyraki^1,7*^‡.

Two supplemental figures and attached to document eight supplemental tables.

- Supplemental Table 1: Primers used in the study.
- Supplemental Table 2: Total protein identified in controls and ERS CMs.
- Supplemental Table 3: List of the predicted classical and non-classical secreted proteins.
- Supplemental Table 4: Significant GO terms of subset of enriched proteins: Molecular Function – Biological Process – Cellular Component – Pathway concerning the 187 classically secreted proteins.
- Supplemental Table S5: List of the secreted proteins found with a higher abundance in ERS CM.
- Supplemental Table S6: List of the secreted proteins found with a lower abundance in ERS CM.
- Supplemental Table S7: Significant GO terms of subset of enriched proteins: Molecular Function – Biological Process – Cellular Component – Pathway – Disease concerning the secreted proteins found with a higher abundance in ERS CM.
- Supplemental Table S8: Significant GO terms of subset of enriched proteins: Molecular Function – Biological Process – Cellular Component – Pathway – Disease concerning the secreted proteins found with a lower abundance in ERS CM.


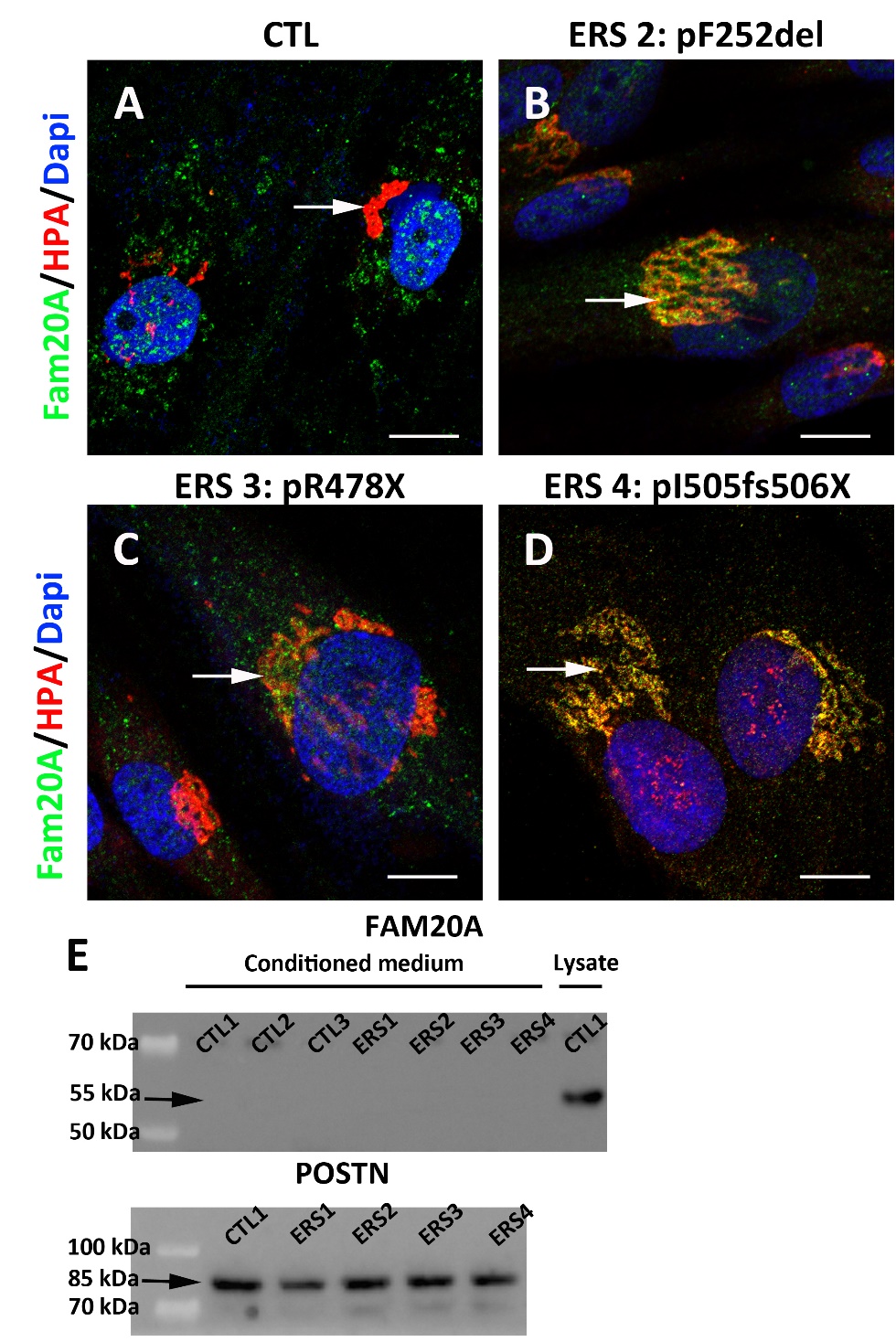


**Supplemental Figure 1**: (A-D) Distribution of FAM20A in control and ERS gingival fibroblasts. (A) In control, FAM20A immunoreactivity (green) was found in large discoidal vesicles, distinct from the cis-Golgi compartment detected using HPA lectin (arrow; red). (B) In ERS2 GF, FAM20A was closely colocalized with HPA (arrow; yellow). (C) In ERS3 GF, a scattered FAM20A signal was seen in small vesicles surrounding the HPA *cis*-Golgi (arrow). (D) In ERS4 GF, the FAM20A signal mainly colocalized with HPA (arrow). (E) Western blot analyses of the conditioned media of three controls and four ERS patients for FAM20A and Periostin (POSTN). POSTN served as a positive control of secretion. FAM20A was found in the cell lysate of control GF cultures. Scale bars: 3μm.


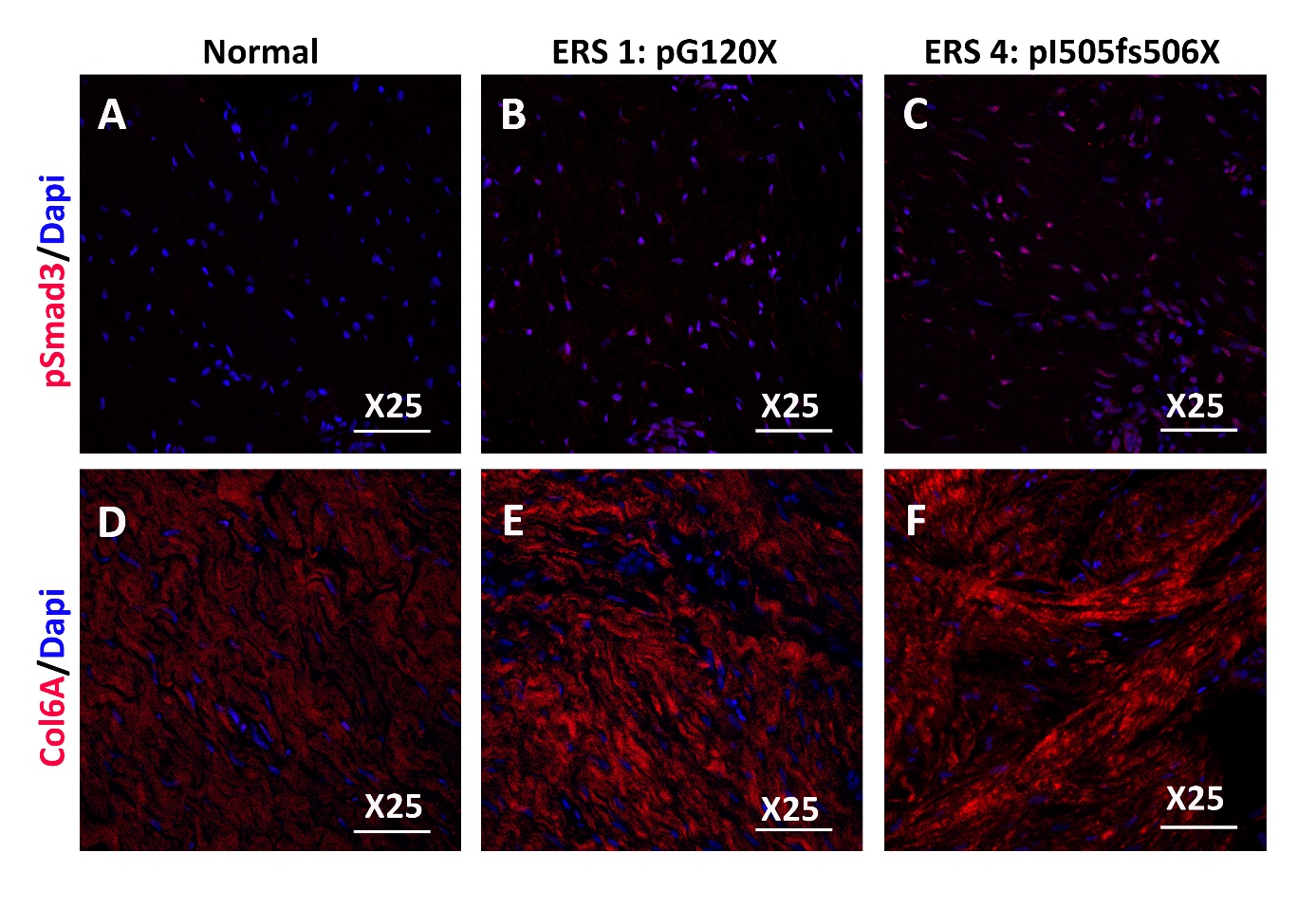


**Supplemental Figure 2**: Low magnifications of gingival tissue from healthy control (A, D) and ERS patients (B, C, E, F) immunostained for p-SMAD3 (A-C) and Collagen VI (D-F). Scale bars: 150 μm.
