# Supplementary material for: Pathogenesis of Enamel-Renal Syndrome Associated Gingival Fibromatosis: A Proteomic Approach
Source: Front Endocrinol (Lausanne). 2021 Oct 29;12:752568. doi: 10.3389/fendo.2021.752568 (PMC8586505; doi:10.3389/fendo.2021.752568)
Supplement: Supplementary file 2 [file Table_1.docx]

**Supplementary Table S1**. List of primers used in the study

| Gene | GenBank accession | Primers | Position | Amplicon |
| --- | --- | --- | --- | --- |
| ANXA2 | NM_001002858 | GAGCGGGATGCTTTGAACATT  TAGGCGAAGGCAATATCCTGT | 160-180  278-258 | 119 |
| BGN | NM_001711 | GAGACCCTGAATGAACTCCACC  CTCCCGTTCTCGATCATCCTG | 685-706  815-795 | 131 |
| CALU | NM_001219.5 | CAGATGATGGTTAGAGATGAGCG  CTCTGTCTTTACCATTCTGGC | 451-473  699-678 | 249 |
| CO6A2 | NM_058174 | TACGGAGAGTGCTACAAGGTG  GGTCCTGGGAATCCAATGGG | 727-747  896-877 | 170 |
| CO6A3 | NM_057166 | CATAACCGCTGTGCGGAAAT  TCATCTAGGGACTTACCACCTG | 303-323  470-449 | 168 |
| CO8A1 | NM_020351 | GCTGCCACCTCAAATTCCTC  CTTTCTTGGGTACGGCTTCCT | 105-124  322-302 | 218 |
| EDIL3 | NM_005711 | TGAGTGCCCAGGCGAATTTAT  ACGTGCATAGTAGGGATACCATT | 429-449  585-563 | 157 |
| FN | NM_212482 | CGGTGGCTGTCAGTCAAAG  AAACCTCGGCTTCCTCCATAA | 125-143  254-234 | 130 |
| GREM1 | [NM_013372](http://www.ncbi.nlm.nih.gov/entrez/query.fcgi?cmd=Search&db=Nucleotide&term=NM_013372) | TCATCAACCGCTTCTGTTACG  GGCTGTAGTTCAGGGCAGTT | 338-358  488-469 | 151 |
| MMP2 | NM_004530 | GATACCCCTTTGACGGTAAGGA  CCTTCTCCCAAGGTCCATAGC | 542-563  653-633 | 112 |
| PTX3 | NM_002852 | CATCTCCTTGCGATTCTGTTTTG  CCATTCCGAGTGCTCCTGA | 4-26  168-150 | 165 |
| SERPINE1 | NM_001165413 | AGTGGACTTTTCAGAGGTGGA  GCCGTTGAAGTAGAGGGCATT | 393-413  543-523 | 151 |
| STC1 | NM_003146 | CACGAGCTGACTTCAACAGGA  GGATGTGCGTTTGATGTGGG | 620-640  729-710 | 110 |
| TGFB2 | NM_001135599 | CCCCGGAGGTGATTTCCATC  GGGCGGCATGTCTATTTTGTAAA | 191-210  330-308 | 140 |
|  |  |  |  |  |
