# Supplementary material for: Pathogenesis of Enamel-Renal Syndrome Associated Gingival Fibromatosis: A Proteomic Approach
Source: Front Endocrinol (Lausanne). 2021 Oct 29;12:752568. doi: 10.3389/fendo.2021.752568 (PMC8586505; doi:10.3389/fendo.2021.752568)
Supplement: Supplementary file 6 [file Table_5.pdf]

Accession	Description	Max fold change
CO8A1_HUMAN	Collagen alpha-1(VIII) chain	15.4
GPNMB_HUMAN	Transmembrane glycoprotein NMB	13.4
HPLN1_HUMAN	Hyaluronan and proteoglycan link protein 1	12.2
NET1_HUMAN	Netrin-1	11.4
TGBR3_HUMAN	Transforming growth factor beta receptor type 3	7.1
CADH6_HUMAN	Cadherin-6	6.7
TGFB2_HUMAN	Transforming growth factor beta-2	6.5
GREM1_HUMAN	Gremlin-1	5.6
MASP1_HUMAN	Mannan-binding lectin serine protease 1	4.4
NPC2_HUMAN	Epididymal secretory protein E1	4.3
MRC2_HUMAN	C-type mannose receptor 2	4.2
PTN_HUMAN	Pleiotrophin	4.1
CCD80_HUMAN	Coiled-coil domain-containing protein 80	4.0
ANGL2_HUMAN	Angiopoietin-related protein 2	3.9
OBAS1_HUMAN	Putative uncharacterized protein OBSCN-AS1	3.9
SEPR_HUMAN	Prolyl endopeptidase FAP	3.9
QSOX1_HUMAN	Sulfhydryl oxidase 1	3.8
PTPRS_HUMAN	Receptor-type tyrosine-protein phosphatase S	3.8
DKK3_HUMAN	Dickkopf-related protein 3	3.8
IBP7_HUMAN	Insulin-like growth factor-binding protein 7	3.6
TSP2_HUMAN	Thrombospondin-2	3.5
DPP2_HUMAN	Dipeptidyl peptidase 2	3.5
GNS_HUMAN	N-acetylglucosamine-6-sulfatase	3.3
NID2_HUMAN	Nidogen-2	3.2
STC1_HUMAN	Stanniocalcin-1	3.2
PDGFD_HUMAN	Platelet-derived growth factor D	3.2
SRPX_HUMAN	Sushi repeat-containing protein SRPX	3.0
PTK7_HUMAN	Inactive tyrosine-protein kinase 7	3.0
1433F_HUMAN	14-3-3 protein eta	2.9
HEXB_HUMAN	Beta-hexosaminidase subunit beta	2.9
TFR1_HUMAN	Transferrin receptor protein 1	2.9
TB182_HUMAN	182 kDa tankyrase-1-binding protein	2.9
TIMP2_HUMAN	Metalloproteinase inhibitor 2	2.8
MMP2_HUMAN	72 kDa type IV collagenase	2.8
TPP1_HUMAN	Tripeptidyl-peptidase 1	2.8
B4GT1_HUMAN	Beta-1,4-galactosyltransferase 1	2.7
PTPRK_HUMAN	Receptor-type tyrosine-protein phosphatase kappa	2.7
SVEP1_HUMAN	Sushi, von Willebrand factor type A, EGF and pentraxin domain-containing protein 1	2.7
CO6A2_HUMAN	Collagen alpha-2(VI) chain	2.7
CO6A3_HUMAN	Collagen alpha-3(VI) chain	2.7
EDIL3_HUMAN	EGF-like repeat and discoidin I-like domain-containing protein 3	2.7
NTRI_HUMAN	Neurotrimin	2.6
CALU_HUMAN	Calumenin	2.6
COEA1_HUMAN	Collagen alpha-1(XIV) chain	2.5
CEMIP_HUMAN	Cell migration-inducing and hyaluronan-binding protein	2.5
CD109_HUMAN	CD109 antigen	2.4
NUCKS_HUMAN	Nuclear ubiquitous casein and cyclin-dependent kinase substrate 1	2.4
SPHM_HUMAN	N-sulphoglucosamine sulphohydrolase	2.3
PPGB_HUMAN	Lysosomal protective protein	2.3
CATZ_HUMAN	Cathepsin Z	2.2
FINC_HUMAN	Fibronectin	2.2
PPIC_HUMAN	Peptidyl-prolyl cis-trans isomerase C	2.2
PGBM_HUMAN	Basement membrane-specific heparan sulfate proteoglycan core protein	2.2
AMD_HUMAN	Peptidyl-glycine alpha-amidating monooxygenase	2.2
CBPQ_HUMAN	Carboxypeptidase Q	2.1
4F2_HUMAN	4F2 cell-surface antigen heavy chain	2.1
DAG1_HUMAN	Dystroglycan	2.1
LUM_HUMAN	Lumican	2.1
ASAH1_HUMAN	Acid ceramidase	2.0
PGS2_HUMAN	Decorin	2.0
CSTN1_HUMAN	Calsyntenin-1	1.9
PEDF_HUMAN	Pigment epithelium-derived factor	1.9
BTD_HUMAN	Biotinidase	1.9
RENR_HUMAN	Renin receptor	1.9
PLOD3_HUMAN	Procollagen-lysine,2-oxoglutarate 5-dioxygenase 3	1.8
BMP1_HUMAN	Bone morphogenetic protein 1	1.7
TUT7_HUMAN	Terminal uridylyltransferase 7	1.7
NUCB1_HUMAN	Nucleobindin-1	1.6
PLOD1_HUMAN	Procollagen-lysine,2-oxoglutarate 5-dioxygenase 1	1.5
