# Supplementary material for: Pathogenesis of Enamel-Renal Syndrome Associated Gingival Fibromatosis: A Proteomic Approach
Source: Front Endocrinol (Lausanne). 2021 Oct 29;12:752568. doi: 10.3389/fendo.2021.752568 (PMC8586505; doi:10.3389/fendo.2021.752568)
Supplement: Supplementary file 7 [file Table_6.pdf]

Accession	Description	Max fold change
ESM1_HUMAN	Endothelial cell-specific molecule 1	15.4
H12_HUMAN	Histone H1.2	5.3
VINC_HUMAN	Vinculin	4.3
PTX3_HUMAN	Pentraxin-related protein PTX3	4.2
CO9_HUMAN	Complement component C9	4.0
H15_HUMAN	Histone H1.5	3.8
CAVN1_HUMAN	Caveolae-associated protein 1	3.7
IF5A1_HUMAN	Eukaryotic translation initiation factor 5A-1	3.5
ISLR_HUMAN	Immunoglobulin superfamily containing leucine-rich repeat protein	3.5
CPNE3_HUMAN	Copine-3	3.3
ANXA6_HUMAN	Annexin A6	3.2
TCPB_HUMAN	T-complex protein 1 subunit beta	3.1
RL13_HUMAN	60S ribosomal protein L13	3.0
HNRPM_HUMAN	Heterogeneous nuclear ribonucleoprotein M	2.9
STOM_HUMAN	Erythrocyte band 7 integral membrane protein	2.6
DDX17_HUMAN	Probable ATP-dependent RNA helicase DDX17	2.6
PTGR1_HUMAN	Prostaglandin reductase 1	2.5
SFRP2_HUMAN	Secreted frizzled-related protein 2	2.4
LOXL3_HUMAN	Lysyl oxidase homolog 3	2.4
TCPG_HUMAN	T-complex protein 1 subunit gamma	2.4
HNRPC_HUMAN	Heterogeneous nuclear ribonucleoproteins C1/C2	2.3
SEPT2_HUMAN	Septin-2	2.3
PAI1_HUMAN	Plasminogen activator inhibitor 1	2.2
PGS1_HUMAN	Biglycan	2.2
GNAI2_HUMAN	Guanine nucleotide-binding protein G(i) subunit alpha-2	2.2
HNRH1_HUMAN	Heterogeneous nuclear ribonucleoprotein H	2.2
ANXA5_HUMAN	Annexin A5	2.2
CKAP4_HUMAN	Cytoskeleton-associated protein 4	2.1
AAAT_HUMAN	Neutral amino acid transporter B(0)	2.1
RS3_HUMAN	40S ribosomal protein S3	2.1
IF4A1_HUMAN	Eukaryotic initiation factor 4A-I	2.0
TCPA_HUMAN	T-complex protein 1 subunit alpha	2.0
DDX3X_HUMAN	ATP-dependent RNA helicase DDX3X	1.9
PDIA6_HUMAN	Protein disulfide-isomerase A6	1.8
1433E_HUMAN	14-3-3 protein epsilon	1.8
ANXA2_HUMAN	Annexin A2	1.7
PLEC_HUMAN	Plectin	1.7
TCPQ_HUMAN	T-complex protein 1 subunit theta	1.7
GDN_HUMAN	Glia-derived nexin	1.6
LTBP2_HUMAN	Latent-transforming growth factor beta-binding protein 2	1.5
